# Apolipoproteins: New players in cancers

**DOI:** 10.3389/fphar.2022.1051280

**Published:** 2022-11-25

**Authors:** Yingcheng He, Jianrui Chen, Yanbing Ma, Hongping Chen

**Affiliations:** ^1^ Department of Histology and Embryology, Medical College of Nanchang University, Nanchang, Jiangxi, China; ^2^ Medical Department, Queen Mary School, Nanchang University, Nanchang, Jiangxi, China; ^3^ Jiangxi Key Laboratory of Experimental Animals, Nanchang University, Nanchang, Jiangxi, China

**Keywords:** apolipoproteins, cancer mechanism, cancer biomarker, anti-cancer therapy, genetic polymorphism

## Abstract

Apolipoproteins (APOs), the primary protein moiety of lipoproteins, are known for their crucial role in lipid traffic and metabolism. Despite extensive exploration of APOs in cardiovascular diseases, their roles in cancers did not attract enough attention. Recently, research focusing on the roles of APOs in cancers has flourished. Multiple studies demonstrate the interaction of APOs with classical pathways of tumorigenesis. Besides, the dysregulation of APOs may indicate cancer occurrence and progression, thus serving as potential biomarkers for cancer patients. Herein, we summarize the mechanisms of APOs involved in the development of various cancers, their applications as cancer biomarkers and their genetic polymorphism associated with cancer risk. Additionally, we also discuss the potential anti-cancer therapies by virtue of APOs. The comprehensive review of APOs in cancers may advance the understanding of the roles of APOs in cancers and their potential mechanisms. We hope that it will provide novel clues and new therapeutic strategies for cancers.

## Introduction

Apolipoproteins (APOs) are proteins that carry lipids, constituting the protein part of lipoproteins. In humans, there are 11 subgroups of APO members including ApoA, ApoB, ApoC, ApoD, ApoE, ApoF, ApoH, ApoL, ApoM, ApoO and ApoJ. Different family members of APOs may interact with distinctive types and amount of lipids to create lipoproteins of various densities (Mehta and Shapiro, 2022). As lipid carriers, the primary function of APOs is transporting lipids. Due to their amphipathic structures, APOs, together with other molecules such as phospholipids and cholesterol, surround lipid cores to form water soluble lipoprotein particles with polar surface (Saito et al., 2004). In this way, lipid components can be transported in the blood circulation. Apart from lipid transport, APOs are also involved in lipoprotein metabolism (Ryan and van der Horst, 2000). Specifically, APOs bind to lipoprotein receptors and other lipid transporters, playing a vital role in lipoprotein utilization and clearance (Ryan and van der Horst, 2000).

Owing to their indispensable role in lipid transport and metabolism, APOs have been extensively explored in the aspect of cardiovascular biology and atherosclerotic disease (Mehta and Shapiro, 2022). Besides, scientists have also found an association between APOs and neurological diseases, and tremendous efforts have been performed in investigation of the function of ApoE in Alzheimer’s disease (Serrano-Pozo et al., 2021). Although APOs in these diseases were well investigated, the roles of APOs in cancer development did not attract enough attention. In fact, early in 1981, rich APOs produced by human hepatoma cell lines were discovered (Zannis et al., 1981). Subsequently, APOs were found to be potential biomarkers in the diagnosis and prognosis of numerous malignancies such as lung cancer, gastric cancer and colorectal cancer, indicating the role of APOs in tumorigenesis and cancer progression (Ko et al., 2014; Quan et al., 2017; Wang et al., 2019a). In recent years, mounting evidence has demonstrated that APOs participate in classical pathways of cancers, encompassing PI3K/Akt, MAPK and Wnt signalings (Jiang et al., 2021a; Wang et al., 2021a; Zhang et al., 2022a). APOs regulate cancer development *via* modulating the hallmarkers of cancers such as apoptosis resistance, inflammation promotion, angiogenesis induction, metastasis activation and proliferation sustainability (Ren et al., 2019a). Based on the mechanisms of APOs in cancer progression, it has also been explored the novel therapeutic potentials of these proteins against tumors (Delk et al., 2021).

As APOs are a crucial player in cancers, there is an urgent need to present a clear view of the relationship between APOs and cancers. Our review concentrated on the roles of APOs in the tumorigenesis and progression of various cancers. We also summarized the current evidence of APOs as biomarkers in different cancers, their genetic polymorphism in conferring cancer risks and possible anti-tumor treatments.

## Structures and physiological functions of APOs

APOs are a set of specialized proteins with the structure of different amino acid sequences encoded by specific genes to constitute lipoproteins together with lipids, which are classified as insoluble APOs (ApoB) and soluble APOs (mainly including ApoA, ApoC, and ApoE) (Mahley et al., 1984; Mehta and Shapiro, 2022). Insoluble APOs play a vital role as a scaffold to assemble neutral and polar lipid molecules as lipoprotein particles for the transportation and redistribution of lipids among various tissues (Dominiczak and Caslake, 2011). Soluble APOs possess the periodic amphipathic α-helix featuring interior repeat units of 11 or 22 amino acids to establish a hydrophobic side for association with internal lipids and a hydrophilic surface exposed to physiological fluids, stabilizing lipoprotein particles and facilitating the interaction between protein and lipid (Segrest et al., 1992).

Different types of APOs perform distinctive functions. ApoA-I is a type of soluble APOs as the major structural component of high-density lipoprotein (HDL). It is secreted by hepatocytes and intestinal cells as a pro-protein, which is converted to nascent HDL after lipidation with the presence of ATP-binding cassette transporter A1 (ABCA1). Then lecithin cholesterol acyltransferase (LCAT) facilitates the unidirectional transformation of discoidal into spherical configuration, converting free cholesterol into cholesteryl ester (CE) (Glomset, 1968; Oram and Vaughan, 2000). ApoA-IV composes chylomicrons, very low-density lipoproteins (VLDLs) and HDLs, involved in the absorption and metabolism of lipid for anti-atherosclerosis effects (Qu et al., 2019). As a primary member of insoluble APOs, ApoB forms the structural backbone of VLDL, IDL, LDL, and chylomicrons, contributing to intravascular transport of lipids as organizing protein (Mehta and Shapiro, 2022). It is synthesized in the endoplasmic reticulum of hepatocytes to produce triglyceride (TG)- rich VLDLs (Mehta and Shapiro, 2022). After the metabolism of VLDLs into CE-rich low-density lipoprotein (LDL), remaining specific amino acids of ApoB interact with intimal proteoglycans and initiate the intention of LDL particles in the arterial wall, which are oxidized and induce local inflammatory and immune responses (Borén et al., 1998; Yurdagul et al., 2016). In addition, lipoprotein metabolism is regulated by soluble APOs as cofactors through controlling the activity of crucial enzymes and by interacting with lipoprotein receptors (McIlhargey et al., 2003). As a soluble APO comprising 79 amino acids, ApoC functions as a crucial activator of lipoprotein lipase (LPL) to stimulate the lipolysis of TG contained in TG-rich lipoproteins (TRLs) by expediting the introduction of TG to the active enzyme site (McIlhargey et al., 2003; Wolska et al., 2017). ApoD is a soluble plasma lipoprotein carrier for the unidirectional lipid transport between cells in the same organ, and between perivascular cells and blood to achieve the homeostasis of many organs (Weech et al., 1991). ApoE as a major soluble APO functioning in the central nervous system (CNS) with 299 amino acids, stimulates the TRL remnant uptake in hepatocytes through binding to the low-density lipoprotein receptor (LDLR) and LDL-related protein 1 (LRP1). However, the interaction between ApoE and its receptors is displaced by ApoC- III, inhibiting the hepatic uptake of remnant particles (Huang and Mahley, 2014; Mahley, 2017). ApoF impacts on lipoprotein metabolism in plasma as a natural preferential inhibitor of cholesteryl ester transfer protein (CETP) activity with LDL to raise the level of HDL cholesterol (Liu and Morton, 2020). ApoM is not only a novel APO that affects both lipoprotein and cholesterol metabolism by improving pre-β HDL formation, but also a chaperone for Sphingosine-1-Phosphate (S1P) for beneficial effects (Ren et al., 2015).

## APOs in cancers

### Brain cancer

In the malignancy of brain, glioma has been considered one of the most important tumors, among which glioblastoma (GBM) is featured with the most invasive progression. In the progression of GBM, cancer cell proliferation, migration, and invasion are promoted due to the overexpression of ApoC1 (Zheng et al., 2022). Moreover, KEAP1, a part of the E3 ubiquitin ligase, can be inhibited by ApoC1 to stimulate the translocation of nuclear factor erythroid 2-related factor 2 (NRF2). The upregulation of NRF2 can thus increase the expression of heme oxygenase-1(HO-1) and quinone oxidoreductase 1 (NQO1), thereby repressing the formation of reactive oxygen species (ROS). In addition, ApoC1 and NRF2 can enhance the expression of cystathionine beta-synthase (CBS) to produce more glutathione (GSH) and further upregulate glutathione peroxidase-4 (GPX4), which can also decrease the level of lipid ROS and Fe^2+^ in GBM cells. ApoC1 can suppress the production of ROS and further inhibit ferroptosis to promote tumorigenesis in GBM through KEAP1/NRF2/HO-1 and NQO1 signaling pathway and CBS/GSH/GPX4 axis (Zheng et al., 2022).

Isocitrate dehydrogenase 1(IDH1) mutation in glioma cells leads to an increased level of 24(S)-hydroxycholesterol (24-OHC), which activates liver X receptors (LXRs). The activated LXRs enhances cholesterol efflux and decreases cholesterol influx by upregulating ApoE, resulting in the cholesterol *de novo* synthesis and the alteration of cholesterol homeostasis in IDH-1 mutant glioma cells, which further contributes to glioma progression (Yang et al., 2020a). Furthermore, Liu et al. indicate that SETD2 mutant/IDH wild-type (SETD2-mut/IDH-WT) GBM cells can activate a specific type of high-grade glioma-associated microglia (HGG-AM) by TGF-β1. HGG-AM shows pro-inflammatory property, which is mediated by upregulated ApoE-induced Nod-like receptor protein 1 (NLRP1) inflammasome. The secreted IL-1β from HGG-AM in combination with ApoE-induced NLRP1 inflammasome trigger the inflammation and proliferation of SETD2-mut/IDH-WT GBM cells, ultimately promoting tumorigenesis (Liu et al., 2021). Additionally, increased ApoE and decreased ApoA1 are found in Grade II meningioma than in Grade I and these two APOs play an important role in delivering lipids to tumors and regulate the redistribution of intracellular lipids. This suggested that the two molecules can be the potential biomarker to predict brain tumor progression (Ghantasala et al., 2021).

Genetic polymorphism of APO genes plays a critical role in brain cancer progression. One pilot study shows that the APOE ε4 allele may induce the cognitive decline of patients with glioma who have received radiotherapy ± chemotherapy. This allele may also increase the susceptibility to impairment of attention and executive function in glioma patients (Correa et al., 2018). Likewise, Correa et al. identified that brain tumor patients with APOE ε4 allele may have increased vulnerability to cognitive dysfunction, such as impaired verbal learning and decreased memory and executive function (Correa et al., 2014).

### Thyroid cancer

Thyroid cancer is a common tumor in the endocrine system, and papillary thyroid cancer (PTC) comprises most cases (Huang et al., 2022). In patients with high-risk PTC or poorly-differentiated thyroid cancer, there is an increased number of LDL receptors in the thyroid, which induced a reduced level of LDL cholesterol and ApoB, the essential component for the recognition and uptake of LDL. Subsequently, the level of 3-hydroxy-3-methylglutaryl-CoA reductase and 25-hydroxycholesterol 7-alpha-hydroxylase are decreased and further lead to the production of 27-hydroxycholesterol (27-HC) metabolite (Revilla et al., 2019). Eventually, the increased 27-HC promotes the proliferation, invasion, and metastasis of PTC (Revilla et al., 2019). Huang et al. reported that obesity-associated protein (FTO), a tumor suppressor, can inhibit tumor glycolysis in PTC. The expression of FTO is decreased in PTC, which leads to an increased level of Insulin-like growth factor 2 mRNA-binding protein 2 (IGF2BP2) mediated-N6-methyladenosine (m6A) modification on the APOE gene. Due to m6A modification, the stability of APOE mRNA increases, which triggers the overexpression of ApoE. Furthermore, reduced FTO in combination with m6A modification of APOE can modulate the IL-6/JAK2/STAT3 signaling pathway to promote tumor glycolysis, thereby aggravating the progression of PTC (Huang et al., 2022).

For the application of APOs in the treatment of thyroid cancer patients, a study shows that a high level of ApoA1 is related to a smaller size of PTC in a male cohort, especially in a subgroup aged less than 55-year-old, which suggests that ApoA1 has the potential to be a diagnostic indicator for PTC (Ma et al., 2021). Moreover, using a high throughput gene expression database, high expression of ApoE is found to correlate with a better prognosis of PTC (Nan et al., 2021). ApoE is related to overall survival and disease-free survival significantly. Low expression of ApoE is also associated with the advanced TNM stage, which indicates that ApoE can act as a biomarker to predict PTC progression and prognosis (Jiang et al., 2020). In addition, liver X receptor β (LXRβ) is overexpressed in PTC and induce the expression of its target genes including APOC1, APOC2 and APOE, which may activate lipid metabolism and protein synthesis, thus facilitating cancer cell proliferation. Therefore, LXRβ and its target APO genes can be novel indicators for diagnosis and therapeutics (Jeong et al., 2020).

### Lung cancer

Previous research showed that APOs play a crucial role in the progression of lung cancer. APOs mediate lung tumorigenesis through activating ERK1/2 pathway. A study shows that ApoM, as a S1P carrier, is overexpressed in non-small cell lung cancer (NSCLC) and can upregulate sphingosine 1-phosphate receptor 1 (S1PR1). Through S1P/S1PR1 signaling, ApoM activates the downstream signaling pathways including ERK1/2 and PI3K/AKT pathways, which promote cancer cell proliferation and invasion *in vitro* and tumor growth *in vivo* (Zhu et al., 2018). In addition, Li et al. found that Immunoglobulin-like transcript 3 (ILT3), an immune inhibitory receptor, is highly expressed in lung tissues of patients with NSCLC (Li et al., 2021a). The interaction of ILT3 with ApoE can recruit SH2 domain-containing phosphatase two and Src homology two domain-containing inositol phosphatase 1(SHP2/SHIP1), which activates ERK1/2 to enhance tumor cell invasion and migration *via* facilitating epithelial-mesenchymal transition (EMT), and induce tumor angiogenesis *via* increasing the production of vascular endothelial growth factor A (VEGF-A), respectively (Li et al., 2021a). Besides, as a part of the chylomicron, ApoE is crucial for cholesterol metabolism by transporting lipids/cholesterol between cells *via* blood. This aroused the speculation that the elevated ApoE could provide more cholesterol for the actively growing cancer cells (Wang et al., 2013).

APOs also facilitate lung cancer progression *via* suppressing the immune system. Knockout of ApoE in mice can inhibit lung cancer cell proliferation and metastasis. The underlying mechanism is the increased infiltration of CD8α+ T cells and CD57^+^ NK cells. NK cells play an essential role in tumor inhibition by upregulating triggering receptor expressed on myeloid cell -1 (TREM-1). Due to the lower expression of ApoE, the upregulation of TREM-1 can be induced by the high expression of T-bet, which is involved in the secretion of IFN-γ, perforin, and granzyme to enhance the anti-tumor cytotoxicity mediated by NK cells (Lee et al., 2019). In addition, knockout or suppressed ApoE expression can also downregulate cyclin D1 and its associated proteins, thereby hampering the tumor proliferation and migration (Wang et al., 2013).

APOs also show the potential for the application of diagnosis and prognostic prediction. In a marker phase I trial, ApoC1 in tumor tissue is highly expressed in late-stage lung cancer and the over-expression is positively correlated with interleukin-6 (IL-6), a molecule that can foster lung cancer cell proliferation. ApoC1 is a promising biomarker for diagnosing lung cancer and the underlying relationship between ApoC1 and IL-6, which may be explored to find novel therapies (Ko et al., 2014). ApoA1 and ApoC-III can be utilized to evaluate the efficacy of neoadjuvant chemotherapy before surgery in patients with small cell lung cancer (SCLC). The expression of ApoA1 and ApoC-III can be prognostic biomarkers to predict the recurrence of SCLC. However, they cannot be used to evaluate the SCLC stage and metastasis (Shi et al., 2016). Besides, Shi et al. reported that the expression of ApoA1 in serum is lower in NSCLC patients, which is associated with poorer survival in NSCLC patients before treatment (Shi et al., 2018). Therefore, the level of ApoA1 in serum can be used as a biomarker to predict metastasis and prognosis.

It is found that an increased level of serum ApoE in NSCLC patients is involved in tumor node metastasis (TNM) stages, lymph node invasion and metastasis. The over-expression of ApoE indicates a poor prognosis of NSCLC, which suggests ApoE can be a prognostic biomarker (Luo et al., 2016). Elevated expression of ApoE shows a strong correlation with lung adenocarcinoma (ADC) (Su et al., 2011). Specifically, ApoE can promote cancer cell proliferation and tumor metastasis in patients with lung ADC and thereby it acts as a potential biomarker for predicting lung ADC progression (Su et al., 2011; Liu et al., 2014). Furthermore, ApoE is highly expressed in lung ADC and NSCLC patients with malignant pleural effusion (MPE), which shows the potential of ApoE to be a diagnostic biomarker for MPE (Su et al., 2011; Wang et al., 2013).

ApoE may also be related to smoking. Rice et al. show that current smokers have a higher level of ApoE than never or ex-smokers. ApoE is intensively related to squamous metaplasia of the lung, indicating that ApoE can be a predictive biomarker for the early stage of lung cancer, especially for current smokers (Rice et al., 2015). Additionally, Xu et al. indicate that residual cells that are not removed by cisplatin-based therapy in NSCLC patients develop drug resistance and these cells finally enter into a dormant state. However, the dormant cancer cells can be activated under specific situations and induce metastasis and recurrence of NSCLC. ApoA1 and ApoE are elevated in dormant cancer cell-derived extracellular vesicles and particles (EVPs), which can be a predictive biomarker for cisplatin resistance (Xu et al., 2021).

### Breast cancer

An increasing number of studies have explored the mechanisms of APOs’ anti-tumor effect on breast cancer (BC). El Roz et al. show that the transcription factor LXR can activate macrophages which then produce ApoE, suppressing the proliferation and promoting apoptosis of breast tumor cells (El Roz et al., 2013). The aberrant upregulation of ApoA1 also has a tumor-killing function. The over-expression of ApoA1 can be activated by estrogen receptor α (ERα) which interacts with the estrogen response element in the ApoA1 gene, leading to an H3K27ac-enriched chromatin environment (Wang et al., 2021b). ApoM is downregulated in BC tissues and can hamper the proliferation and invasion of the cancer cells, possibly by upregulating the expression of vitamin D receptors (VDRs) (Zhou et al., 2022). By contrast, ApoC1 is shown to promote breast tumorigenesis. Mechanistically, ApoC1 can increase the expression of vimentin and reduce the expression of E-cadherin, thus contributing to the progression of EMT. Meanwhile, ApoC1 can also suppress the activation of the JNK/MAPK pathway to induce the proliferation of breast cancer cells (Zhang et al., 2022a). It seems that different members of APOs play distinctive roles in BC. However, APOs may also exert their functions depending on the cell line of BC. One study shows that in MDA-MB-231 cells, the expression of ApoE or ApoA1 inhibit the growth and migration of tumor cells *via* preventing EMT, probably through preventing the *de novo* production of fatty acids and cholesterol entry into BC cells. However, the opposite effect is observed in the MCF-7 cell line (Liu et al., 2013; Ben Hassen et al., 2020). Therefore, the underlying mechanism of APOs in promoting or inhibiting breast tumorigenesis requires further investigation.

APOs can also serve as biomarkers for detecting and monitoring BC. Several APOs are closely related to the diagnosis and prognosis of cancer. Compared to healthy individuals, patients with BC have an increased level of ApoE in the serum, which is related to the TNM stage for the assistance of cancer progression. Patients with higher serum ApoE have worse overall survival compared with those with lower ApoE level. Serum ApoE is an independent risk factor for the prognosis of BC patients (Xu et al., 2016). These suggest that serum level of ApoE can serve as a biomarker for disease development and prognostic prediction of BC. An increased level of ApoD can confer an approximately two-fold risk of BC-related death and predict poor survival in these patients independent of the expression of androgen receptor or ERα (Jankovic-Karasoulos et al., 2020). It is demonstrated that ApoA1 and ApoE may act as biomarkers in male BC due to their upregulation in the serum (Zografos et al., 2019). APOs are also associated with metastasis of BC, especially to the eyes. For instance, BC patients with intraocular metastasis (IOM) have significantly increased ApoB and decreased ApoA1 than those without IOM (Liu et al., 2019). Likewise, a reduced level of ApoA1 serves as a risk factor for predicting IOM in patients with postmenopausal BC or invasive ductal carcinoma (Li et al., 2021b; Liang et al., 2021). APOs are indicated as risk factors for predicting BC relapse. Specifically, a recent study constructs an autophagy-related mRNA signature including ApoL1, which is remarkably correlated with early relapse in patients with BC (Min et al., 2022). In addition, an increased level of ApoB is related to a higher incidence of cancer recurrence, but no association is found between the level of ApoA1 and cancer relapse (Harborg et al., 2022).

The relationship between the polymorphism of APOs and BC has been explored. The APOA1 (+83) T allele is related to a higher risk of metastasis of BC to lymph nodes while the (-75) GA genotype is associated with the premenopausal status of patients with BC. Besides, the ApoA1 -75 A allele is positively correlated with risk of BC (Hamrita et al., 2011). In terms of ApoB, it has been found that its polymorphism of rs693 (-7673C>T) and rs1042031 (-12669 G>A) are remarkably associated with an increased risk of BC in Chinese patients (Liu et al., 2013). In addition, the correlation between APOs polymorphism and cancer risk seems to be different among various populations. Specifically, compared to the normal allele ε3, the APOE gene ε4 allele is significantly associated with a higher risk of BC in Asian people, while in Caucasians, there is no such remarkable association (Saadat, 2012).

### Gastric cancer

PI3K/AKT signaling pathway plays a vital role as a classical pathway in various cancers, which is affected by APOs in the progression of gastric cancer (GC). ApoC2 is upregulated in GC tissues with peritoneal metastasis and correlates with a worse prognosis. Mechanistically, through interacting with CD36, ApoC2 induces the activation of the PI3K/AKT/mTOR signaling pathway, promoting the over-expression of EMT markers and confer mesenchymal phenotype of GC cells and eventually foster cancer metastasis. As a fatty acid translocase, CD36 regulates the lipid metabolism of GC cells and its overexpression promoted the fatty acid uptake of the tumor cells. Therefore, it is supposed that ApoC2 satisfies the energy need of tumors and thus help them to metastasize (Wang et al., 2021a). Study also suggests that tumor-associated M2 macrophages in GC can produce exosomes containing functional ApoE, which mediate the transfer of ApoE-stimulated PI3K/AKT signaling pathway to recipient GC cells, resulting in the pro-migratory phenotype of the tumor cells. (Zheng et al., 2018). Additionally, the network of ApoE-associated genes is investigated, including signal transducer and activator of transcription 2 (STAT2) (Shi et al., 2015).

APOs can serve as diagnostic and prognostic biomarkers for GC. It is suggested that ApoA1 and ApoB may help to diagnose and monitor GC. Compared to GC patients at early stage, those at advanced stage have reduced ApoA1 and increased ApoB level (Kimak et al., 2019). A diagnostic score of GC can be formulated based on the signature peptides, such as ApoC1 and ApoC-III, which can differentiate GC patients from healthy individuals. The data show that serum levels are reduced in patients with GC (Cohen et al., 2011). Similarly, the serum levels of ApoC1 and ApoC-III are lower in GC patients compared to patients with gastritis (Wang et al., 2019a). Previous studies have shown that ApoC1 prevents lipoproteins from binding to LDL receptors while ApoC-III hampers the interaction of lipoproteins to glycosaminoglycan on cell surface, both leading to a delayed clearance of triglycerides (Shachter, 2001). This might explain why GC patients often have decreased serum lipid levels (Ghahremanfard et al., 2015). Thus, the low concentration of serum ApoC1 and ApoC-III may contribute to GC diagnosis. However, a following study shows that ApoC1 is up-regulated in serum and GC tissues, which is related to shorter survival of GC patients. Besides, ApoC1 expression is remarkably associated with tumor classification and clinical stage (Yi et al., 2019). These indicate that the expression of ApoC1 in GC remains controversial, but it may act as a biomarker for both diagnosis and prognosis of GC. Preoperative serum ApoB/ApoA1 ratio can also act as an independent factor for GC prognosis and it is the best predictor in all lipid-related factors. A high ApoB/ApoA1 ratio is correlated with poorer survival (Ma et al., 2018).

It has been indicated that specific genetic variants of APOs may confer risk for GC. There are three polymorphic alleles of the APOE gene related to diseases, and one of them, namely APOE ε2, is associated with a high risk for the development of GC in Chinese Han population. The elevated risk may be mediated by decreased serum total cholesterol level, which is a risk factor for GC development. However, the presence of APOE ε2 may not correlate with cancer progression as it is not significantly associated with the cancer stage (Kang et al., 2016a). Intriguingly, the opposite conclusion is drawn from the Caucasian population. Compared to those with two APOE ε3 alleles, approximately 60% reduction of GC is observed in individuals carrying one or two ε2 alleles and this may be partially due to a better antioxidant property of the APOE ε2 allele instead of a diminished cholesterol level (De Feo et al., 2012).

### Liver cancer

As the liver synthesizes the majority of plasma endogenous lipoproteins and lipids, chronic liver diseases affect lipid and apolipoprotein metabolism, which serve as the basis for developing into liver cancer. ApoA1 can suppress hepatocellular carcinoma (HCC) by promoting cell cycle arrest and enhancing tumor cell apoptosis *via* inactivating the mitogen-activated protein kinase (MAPK) signaling pathway. It may also hamper the tumor cells from degrading the extracellular matrix and thus inhibit their dissemination (Ma et al., 2016). Similarly, it is identified that ApoE exerts a tumor-suppressive effect in liver cancer. During the malignancy development of hepatocytes, cadmium can enhance the DNA methylation of the ApoE promoter region and prevent the action of the transcriptional regulator of ApoE called LXRα, thus downregulating the expression of ApoE and leading to tumor invasion (Suzuki et al., 2017). Recently, it has been shown that ApoE can also be inhibited by cadmium-induced downregulation of ten-eleven translocation methylcytosine dioxygenase 1 (TET1) (Hirao-Suzuki et al., 2021). ApoM acts as a tumor suppressor and it modulates glycolysis of liver cancer cells *via* the transcription factor sterol regulatory element-binding protein 1 (SREBP1) pathway. Data have shown that knockout of ApoM can lead to over-expression of SREBP1 and ATP-dependent 6-phosphofructokinase, liver type (PFKL), eventually causing liver tumor proliferation and metastasis (Zhang et al., 2022b). ApoM can also prevent HCC cell proliferation and migration possibly *via* upregulating the expression of VDR (Yu et al., 2019). It has been shown that the downregulation of ApoM reduced the autophagy of hepatocytes, resulting in the accumulation of liver lipids (Bai et al., 2021). Additionally, in hepatoma, ApoL1 expression can be activated by a high level of interferon regulatory factor (IRF) 1, IRF2 and Sp1, all of which are transcription factors that can bind the ApoL1 promoter region (Wang et al., 2020a).

As APOs play a crucial role in liver cancer progression, they can function as diagnostic or prognostic biomarkers. ApoA1 is remarkably reduced in the serum of patients with HCC while C-reactive protein (CRP) is significantly increased. Both of these markers are associated with shorter disease-free survival and overall survival (Mao et al., 2018). A low level of ApoA1 can also independently predict the early recurrence of HCC (Ni et al., 2020). Thus, the AC score is designed, which is the combination of ApoA1 and CRP, and is an accurate predictor for HCC prognosis (Mao et al., 2018). ApoA1 can also be analyzed with neutrophils, forming a neutrophil-to-ApoA1 ratio (NAR), which can predict the overall survival of HCC patients treated with transarterial chemoembolization (Chen et al., 2021). Besides, in hepatitis B virus-related HCC, ApoA1 and ApoC1 are potential diagnostic markers while ApoC3 and ApoC4 are likely to be both diagnostic and prognostic biomarkers (Wang et al., 2019b). Early in 2009, it is found that HCC tissues expressed a lower level of ApoM than adjacent tissues but the plasma level of ApoM was elevated in HCC patients compared to normal individuals (Jiang et al., 2011). This indicates that those healthy liver tissues or extra-hepatic tissues produce more ApoM (Jiang et al., 2011). Experiment *in vivo* shows that the deletion of ApoM can accelerate the development of mutations in liver cancer cells induced by N-nitrosodiethylamine, a carcinogenic agent (Bai et al., 2021). Therefore, ApoM may be a protective factor to prevent primary liver cancer. Different from ApoA1 and ApoM, a high ApoB level may be a risk factor for HCC as it is correlated with poorer post-surgery survival in HCC patients, and individuals with higher ApoB level tend to have a larger tumor size. Besides, majority of LDL bind to LDLR *via* ApoB on its surface and then ApoB is degraded by the liver. This indicates that the increased ApoB serum level may reflect downregulation of LDLR (Yan et al., 2019). Some APOs may be related to the clinical characteristics of patients with liver cancer. For example, HCC tissues have downregulated level of ApoF, which is related to the TNM stage and liver cirrhosis. ApoC-III is also remarkably associated with tumor grade and stage (Chang et al., 2019a).

The genetic information of APOs may indicate liver cancer. A study discovered that ApoB is frequently mutated in HCC patients, accounting for nearly 10% of all mutations (Nault et al., 2020). Specifically, a non-oncogenetic mutation of APOB is observed, which can cause APOB inactivation and is correlated with overexpression of oncogenic regulators and downregulation of tumor suppressors, leading to worse outcomes of survival. It is speculated that APOB inactivating mutation is preferred in tumorigenesis to provide more energy to cancer metabolism because much energy is required to form VLDL with ApoB-100, a isoform of ApoB (Lee et al., 2018). Besides, the polymorphism of APOE has been investigated in liver cancer. Innes et al. found that rs429358 of APOE is associated with a decreased risk of developing HCC in patients with liver cirrhosis (Innes et al., 2022). The E4 allele of APOE can function as a protective factor in reducing inflammation and liver fibrosis in HCV-infected patients, which may decrease the risk of liver cancer (Nascimento et al., 2021).

### Pancreatic cancer

Many APOs act as tumor activators in pancreatic cancer (PC). The monocyte-expressed ApoE in peripheral blood is increased in patients with pancreatic ductal adenocarcinoma (PDA). Furthermore, ApoE promotes immune suppression in PDA primarily by stimulating LDLR on cancer cells and thus activates NF-κB signals to induce the production of immunosuppressive factors from tumor cells such as CXCL1 and CXCL5 (Kemp et al., 2021). ApoE can also directly enhance tumor proliferation. It is demonstrated that ApoE2–LRP8 (lipoprotein receptor-related protein 8) pathway is highly activated in PC, which triggers the phosphorylation of ERK1/2 to stimulate c-Myc. The activation of c-Myc suppresses the transcription of p21^Waf1^ and increases the expression of cyclin D1 and B1, which promote the distribution of cell cycle and cell proliferation (Du et al., 2020). Through activating the ERK1/2 pathway, ApoE2 can further promote the expression of MMP-2/9 and EMT, resulting in tumor cell invasion and migration (Wang et al., 2020b). Similar to ApoE2, ApoL1 is also up-regulated in PC tissues and it acts as an oncogene to induce proliferation and prevent apoptosis of the tumor cells *via* stimulating the Notch1 signaling pathway (Lin et al., 2021). By contrast, ApoA1 functions as a tumor suppressor involved in lipid metabolism reprogramming in PC. The tripartite motif (TRIM) family member TRIM15 is overexpressed in PDA tissues, which enhances the expression of LDLR and promotes ApoA1 to be ubiquitinated and degraded. This promoted lipid anabolism and results in increased accumulation of lipid droplets in PC tissues, eventually leading to PC cell proliferation, migration and metastasis. Conversely, an elevated level of ApoA1 can inhibit the expression of LDLR and reverse the effect of TRIM15 (Sun et al., 2021).

ApoA2-ATQ/AT, one of the ApoA2 isoforms, is a promising early biomarker that may play a crucial role in detecting PC prior to imaging. One study indicates that plasma ApoA2-ATQ/AT combined with carbohydrate antigen 19–9 (CA19-9) can help to detect PC up to 18 months before diagnosis under normal care (Honda et al., 2019). Besides, ApoA2-ATQ/AT itself can increase the diagnostic probability and help to discriminate patients with early-stage PC from healthy people and find those at high risk for PC (Honda et al., 2015; Sato et al., 2020). ApoA2-ATQ/AT functions as a marker for identifying pancreatic exocrine disease in PDA patients after chemoradiotherapy (CRT). This is because the ApoA2-ATQ level can be significantly affected by pancreatic enzymes which are secreted insufficiently during the pancreatic exocrine disease (Hayasaki et al., 2019). Individuals with surgically-curable PC can also be identified by detecting serum level of ApoA2-I (Honda, 2022). While ApoA2-ATQ/AT is mainly used to detect early PC, ApoE can be a marker for PC at an advanced stage. The lymph node metastasis and TNM staging are associated with overexpression of ApoE in PC tissues, thus reflecting the tumor progression (Chen et al., 2013a; Chen et al., 2013b). As another member of the APO family, ApoC2 in the serum can predict the survival of PC patients after pancreaticoduodenectomy, and thereby it may help to select PC patients for the surgery (Xue et al., 2012).

### Colorectal cancer

As mentioned, in breast and liver cancer, ApoM can up-regulate the expression of VDR, which may be involved in the anti-neoplastic effect of ApoM. In colorectal cancer (CRC), a study also indicates the similar relationship between ApoM and VDR but the role of ApoM in the cancer remains controversial (Yu et al., 2017). A study shows that ApoM may inhibit CRC development (Yu et al., 2017), while another study suggests that the high expression of ApoM in CRC cells promotes cancer cell proliferation and suppressed apoptosis *via* increasing the expression of ribosomal protein S27A (RPS27A) (Mu et al., 2021). Several APOs are considered as protective factors for CRC. For instance, upregulation of both ApoA1 and ApoA1 binding protein (AIBP) can suppress CRC cell proliferation and metastasis. Moreover, AIBP-ApoA1 fusion protein also shows a synergistic effect against CRC migration and angiogenesis *via* increasing cholesterol efflux and damaging the correct distribution of invasion- and migration-related proteins on the membrane raft (Zhang et al., 2019a). ApoE may prevent the carcinogenesis of CRC as its deletion causes a higher susceptibility to inflammation-related tumorigenesis of CRC (Tanaka et al., 2016). In the process of CRC development, due to DNA methylation of its promoter, the level of ApoD is reduced but it remains the ability to react to oxidative stress. Experiments have demonstrated that in the condition of paraquat-triggered oxidative stress, the exogenous introduction of ApoD to CRC cells enhances tumor apoptosis (Bajo-Grañeras et al., 2013). However, APOs such as ApoC1 may promote CRC progression. It has been shown that ApoC1 level is upregulated in CRC tissues, which is associated with TNM stage and poorer survival, and promotes CRC development probably *via* the MAPK pathway (Ren et al., 2019b). Another research also indicates that upregulation of ApoC1 promotes liver metastasis of CRC (Lu et al., 2021).

In CRC patients, reduced ApoA1 level is related to advanced TNM stage and systemic inflammation, indicating its role in predicting prognosis (Sirniö et al., 2017). In terms of inflammation, the decrease of ApoA1 in CRC patients is accompanied by a reduction of CRP. Thereby it is proposed that the combination of CRP and ApoA1 may improve the clinical staging of CRC patients (Ye et al., 2019). ApoA1 may be a promising biomarker for guiding individualized treatment for CRC patients. It can be used to predict the treatment efficacy of bevacizumab. Specifically, metastatic CRC patients with a lower level of pre-treatment ApoA1 gain less benefit from the drug and have inferior survival (Quan et al., 2017). A negative correlation is found between serum ApoA1 and the response to neoadjuvant chemoradiotherapy in patients with rectal cancer (Guo et al., 2021). Although ApoB is not shown to correlate with tumor stage (Sirniö et al., 2017), it can serve as a predictor of survival in CRC patients after radical surgery (Chen et al., 2020a). Glycated ApoB is found more in CRC and adenoma tissues than in adjacent noncancerous tissues, indicating that it may be related to dysplastic and neoplastic development (Reddavide et al., 2011). In addition, elevated serum ApoB/ApoA1 level predicts a poorer survival in patients with metastatic CRC and also a higher risk of liver metastasis in patients with locally advanced rectal cancer (Yang et al., 2020b; Chen et al., 2022).

The expression level of ApoE is higher in primary CRC with synchronous liver metastasis than that in stage II CRC without progression in 5 years. It is suggested that ApoE over-expression is correlated with CRC progression and shorter survival through three potential ways, including cholesterol and bile metabolism, triglyceride and insulin regulation, and the prolonged inflammation (Zhao et al., 2018). Moreover, the relationship between APOE polymorphism and CRC has also been explored. The APOE ε4 allele is associated with a reduced risk of developing proximal colorectal neoplasia (Tian et al., 2014).

### Renal cell carcinoma

Renal cell carcinoma (RCC) is an important kind of kidney cancer and clear cell renal cell carcinoma (ccRCC) comprises almost 80% of RCC (Guo et al., 2016; Li et al., 2020a). Several studies show that ApoC1 is related to the progression of RCC. Jiang et al. indicate that highly expressed ApoC1 correlate with a poor prognosis of RCC (Jiang et al., 2021a). *In vitro*, ApoC1 promotes not only cancer cell proliferation but also migration and invasion *via* inducing EMT (Jiang et al., 2021a). Furthermore, it is testified that ApoC1 can regulate the WNT3a signaling pathway to facilitate the development of RCC (Jiang et al., 2021a). In terms of ccRCC, it is reported that overexpression of ApoC1 is associated with poor overall survival of patients (Li et al., 2020a). Mechanistically, ApoC1 induces EMT by activating STAT3, eventually promoting metastasis of the tumor (Li et al., 2020a). Moreover, the overexpression of ApoC1 can inhibit the interaction of VLDL with VLDLR, resulting in the decreased uptake of VLDL and post-lipolysis particles, which may be the potential mechanism leading to poor clinical prognosis of ccRCC (Cui et al., 2020).

Distinctive from ApoC1, ApoB and ApoA1 seem to be tumor suppressors in RCC. The ApoB level can reflect the malignancy degree of ccRCC. A high level of ApoB can improve the survival rate of patients with ccRCC before surgery, which is valuable to be used to evaluate prognosis and survival rate in ccRCC patients (Wang et al., 2020c). The underlying reason is that the level of cholesterol is positively related to the cancer progression. Thus, due to the negative feedback regulation of cholesterol synthesis, there is diminished synthesis of endogenous cholesterol, which further leads to a lower level of LDL, ultimately causing reduction in ApoB (Wang et al., 2020c). Likewise, an increased level of pretherapy ApoA1 is engaged in better prognosis in surgical RCC patients (Guo et al., 2016). Intriguingly, in patients with metastatic renal cell carcinoma (mRCC), a high ratio of preoperative ApoB/A1 indicates poor progression-free survival and overall survival (Zhang et al., 2019b). These studies suggest that it is noteworthy to further explore the mechanism of ApoB and ApoA1 in RCC, which can be promising biomarkers to predict prognosis and improve the effectiveness of treatment.

### Bladder cancer

Previous studies showed that APOs play a vital role in the diagnosis and prognostic prediction of bladder cancer. It is reported that the elevated level of ApoA1 in urine can facilitate the diagnosis of patients with bladder cancer, which shows high sensitivity and specificity (Li et al., 2014). Shang et al. discovered that the presence of a high preoperative level of ApoA1 in the blood can improve overall survival and cancer-specific survival in patients with non-muscle invasive bladder cancer (NMIBC). ApoA1 can be a valuable indicator for patients who will receive surgical treatment and help to choose a better therapeutic regimen (Shang et al., 2018). In addition, it is showed that the genetic variants of APOA1 are associated with bladder cancer. Specifically, bladder cancer cases with APOA1-75 AA genotype reveal a high ApoA1 level in urine. Furthermore, urinary ApoA1 in low-grade bladder cancer cases shows a high expression, while patients with high-grade bladder cancer are more likely to have a low urinary ApoA1 expression. Therefore, APOA1-75 AA variants in combination with ApoA1 expression may act as a valuable tool to evaluate the malignant degree of bladder cancer (Magray et al., 2021).

The urine level of ApoA1 and ApoA2 also have the potential for predicting bladder cancer. ApoA1 can be utilized for early detection whereas ApoA2 can identify tumor stages (Chen et al., 2010). Through analysis of extracellular vesicles in urine, Andreu et al. demonstrated that the existence of ApoB in 100,000 g pellet can be a biomarker for diagnosing bladder cancer (Andreu et al., 2017). The detection of ApoB plays a vital role in the diagnosis and discrimination of the grade of bladder cancer (Andreu et al., 2017).

### Prostate cancer

Studies have been carried out to explore the role of APOs in prostate cancer. Su et al. found that ApoC1 can boost prostate cancer cell proliferation by upregulating the expression of Survivin. As an anti-apoptotic factor, Survivin can accelerate the cell cycle and inhibit apoptosis of cancer cells *via* Survivin/Rb/p21/caspase-3 signaling pathway (Su et al., 2018). Over-expression of ApoA1 in prostate cancer can result from the aberrant metabolism of lipids, which enhances cell proliferation, invasion and induces resistance to the hormonal treatment. Moreover, it is also indicated that up-regulation of ApoA1 can be induced by over-expressed MYC in prostate cancer cells, which suggests a possible reason for the aggravation of the disease. This finding reveals that ApoA1 has the potential to be a diagnostic and prognostic indicator for the progression of prostate cancer (Wang et al., 2021c). Nevertheless, a Swedish Apolipoprotein Mortality Risk (AMORIS) study shows a different result, which is that a low level of ApoA1 and HDL may be a risk factor for prostate cancer patients. The underlying mechanism may be that decreased HDL and ApoA1 can result in the inflammation and further contribute to the prostate cancer progression (Van Hemelrijck et al., 2011). The distinction suggests that more experiments require to be carried out to figure out the mechanism of ApoA1 in prostate cancer.

The polymorphism of APOE may play a significant role in prostate cancer. For the African-American population, Ifere et al. divided weakly, moderately and highly tumorigenic prostate cancer cell lines into two groups: non-aggressive (weakly tumorigenic) and aggressive (moderate and highly tumorigenic). They utilized these cell lines to explore the influence of genetic variants of APOE on prostate cancer. They discovered that APOE ε3/ε3 or ε3/ε4 alleles were present in non-aggressive cell lines while APOE ε2/ε4 alleles were present in aggressive cell lines. Aggressive cell lines showed a dysregulated cholesterol efflux and deposit at the membrane in contrast to non-aggressive cell lines (Ifere et al., 2013). Moreover, in the Turkish population, the ε3/ε3 genotype is probably a risk factor for prostate cancer and patients of this genotype may have a high Gleason score. On the contrary, the ε4 allele is a risk reduction factor for prostate cancer (Yencilek et al., 2016).

### Cervical cancer

Cervical cancer is one of the most malignant tumors in the female reproductive system. It is demonstrated that ApoA1 mediates the resistance to platinum-based chemotherapy in patients with cervical cancer. In mechanism, ApoA1 can promote tumor growth and metastasis by STAT1/p38 MAPK signaling pathway, enhance cancer progression by CD81/C3/PI3K signaling pathway, and induce recurrence by TOP2A (He et al., 2022a). Up-regulation of ApoC1 can promote cervical cancer cell growth both *in vitro* and *in vivo*. Besides, ApoC1 is engaged in metastasis by inducing EMT *in vivo*. Knockdown of the APOC1 gene can inhibit cancer progression, which indicates that ApoC1 has the potential to be a target for diagnosis and therapeutics (Shi et al., 2020). In addition, it is reported that decreased level of pretherapy ApoC-II in serum can lead to a poor prognosis in patients who have received chemoradiotherapy (Harima et al., 2013). Cervical cancer patients with a pretherapy level of ApoC-II ≤25.8 μg/ml have shorter pelvic progression-free survival (PPFS) than those with a pretherapy level of ApoC-II >25.8 μg/ml (Harima et al., 2021), which indicates that ApoC-II can be a useful biomarker for patients with cervical cancer to predict prognosis and assist clinicians in choosing a better regimen, which ensures the best effect of treatment.

**FIGURE 1:**
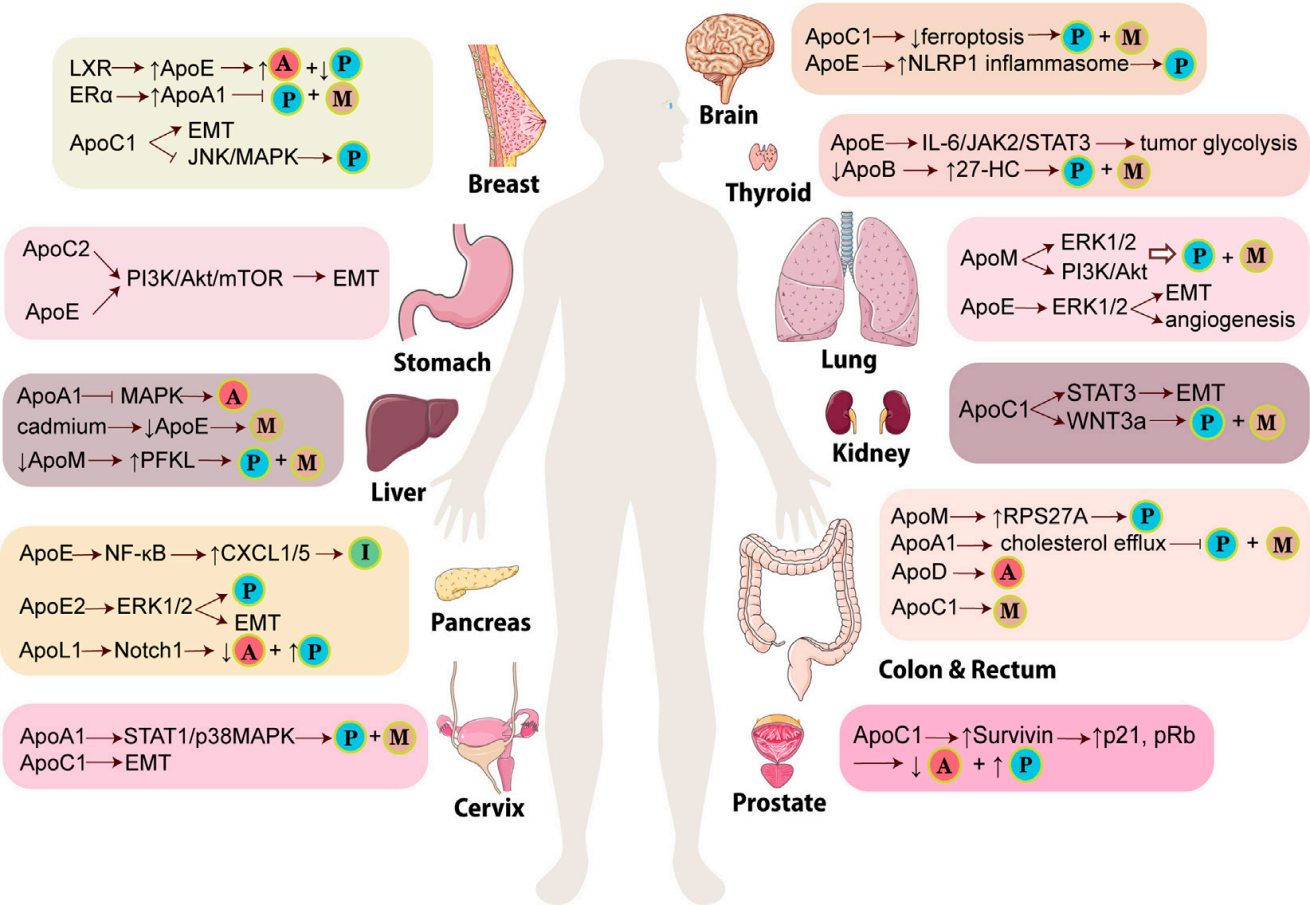
Mechanisms of APOs participating in the development of various cancers. In different types of cancers, APOs promote or inhibit tumor *via* different signaling pathways and lead to various results of tumors. The little balls indicate the critical events of cancers induced or prevented by APOs. The ball with “P” means tumor proliferation while the ball with “M” means tumor metastasis, migration or invasion. The ball with “A” means tumor apoptosis whereas the ball with “I” means immune suppression. (LXR: liver X receptor; ERα: estrogen receptor α; PFKL: ATP-dependent 6-phosphofructokinase, liver type; CXCL: C-X-C motif chemokine ligand; EMT: epithelial-mesenchymal transition; NLRP1 inflammasome: Nod-like receptor protein one inflammasome; 27-HC: 27-hydroxycholesterol; RPS27A: ribosomal protein S27A). This figure is partially made using Servier Medical Art (smart.servier.com).

**FIGURE 2:**
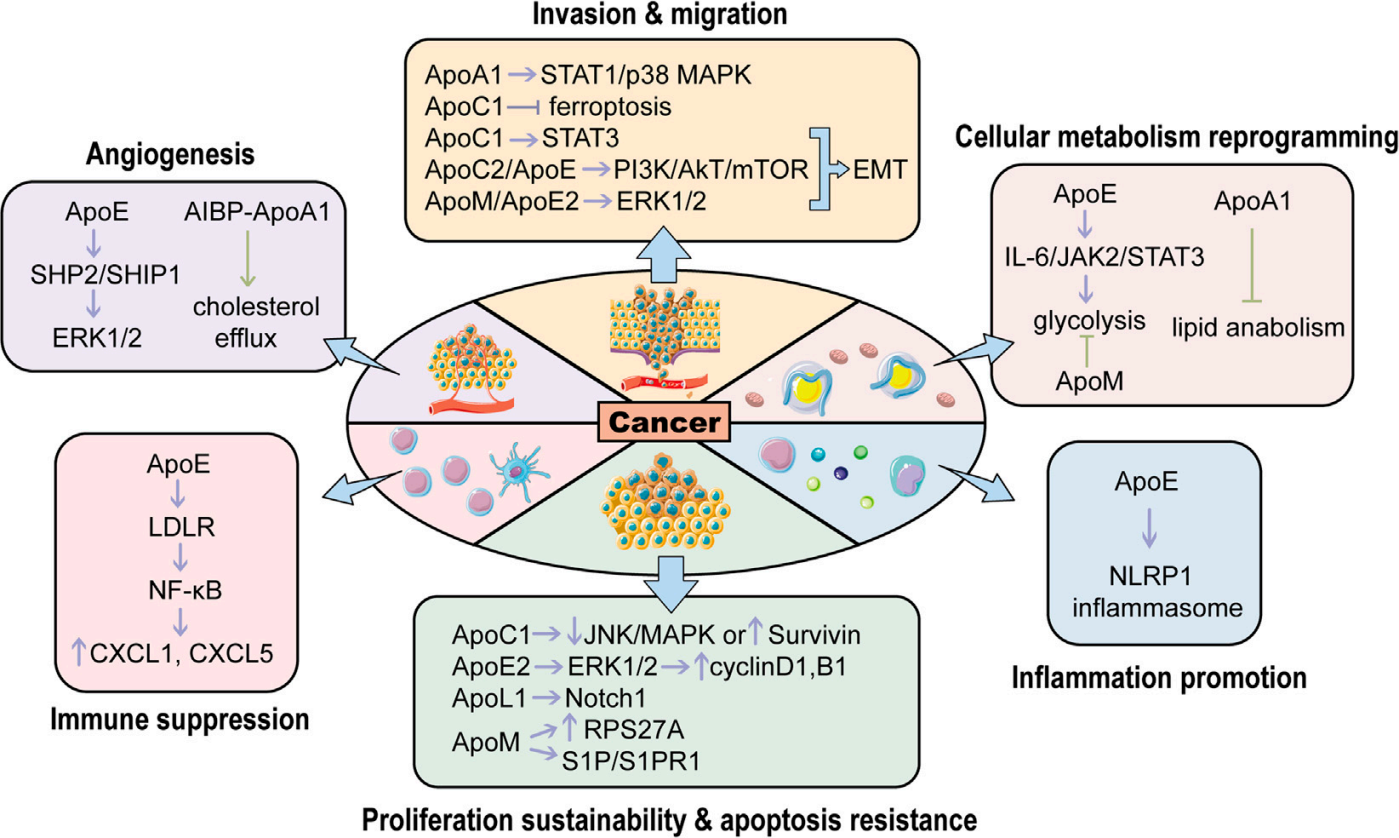
The roles of APOs in regulating various hallmarks of cancers. APOs activate or inhibit carcinogenesis through modulating different hallmarks of cancers. The purple arrows represent cancer promotion while the green ones represent cancer suppression. (EMT, epithelial-mesenchymal transition; NLRP1 inflammasome: Nod-like receptor protein one inflammasome; RPS27A, ribosomal protein S27A; S1P, Sphingosine-1-Phosphate; S1PR1, sphingosine 1-phosphate receptor one; LDLR, low-density lipoprotein receptor; SHP2: SH2 domain-containing phosphatase two; SHIP1, Src homology two domain-containing inositol phosphatase one; AIBP, ApoA1 binding protein). This figure is partially made using Servier Medical Art (smart.servier.com).

To sum up, APOs are involved in multiple types of cancers (Figure 1). From one perspective, these proteins modulate the classical signaling pathways in cancers, such as PI3K/Akt, MAPK and Wnt signalings, to influence tumor initiation and progression. From another, APOs also interact with components around tumors such as immune cells, thereby regulating the cellular response to cancers. Overall, APOs serve as tumor promoters or suppressors in modulating various hallmarks of cancers, which mainly encompass proliferation sustainability, apoptosis resistance, inflammation promotion, cellular metabolism reprogramming, angiogenesis, immune suppression, invasion and migration (Figure 2). Therefore, they are potential biomarkers for diagnosis and prognosis for cancers and the understanding of the mechanism of APOs in cancers is conducive to establishing related anti-tumor treatments. Apart from the cancer types mentioned above, APOs are also indicated in the others cancer with relatively fewer attention (Table 1).

**TABLE 1:** The participation of APOs in other types of cancers.

Type of cancer	Type of apolipoproteins	Biological mechanism	Biomarker or genetic polymorphism	Ref
melanoma	ApoE	ApoE ↑	APOE4	Pencheva et al. (2012); Ostendorf et al. (2020)
		(-) invasion and angiogenesis	(-) melanoma progression	
		(-) metastatic endothelial recruitment	(-) tumor metastasis	
			(+) anti-tumor immune effect	
			(+) survival rate	
			APOE2	
			(+) melanoma progression	
			(+) tumor metastasis	
			(-) anti-tumor immune effect	
			(-) survival rate	
pituitary adenoma	ApoE		APOE E2/E3 allele	Sidaraite et al. (2019)
			(+) tumor initiation	
			APOE E2/E4 allele	
			(+) tumor development	
			APOE E4/E4 allele	
			(+) tumor recurrence	
			APOE E3/E3 allele	
			(-) tumor progression	
head and neck cancer	ApoE ApoL1	ApoL1 ↑: (+) tumor progression	APOE E2 allele	De Feo et al. (2010); Zhong et al. (2020)
			(+) antioxidant effect	
			(-) tumor progression	
			ApoL1	
			diagnostic biomarker	
oral squamous cell carcinoma	ApoL1 ApoE	ApoL1 ↑	ApoL1: diagnostic biomarker	Jayakar et al. (2017); Zhong et al. (2020)
		(+) tumor progression		
		ApoE ↑		
		(+) ECM degradation		
		(+) Phosphorylation of		
		ERK, c-Jun, and JNK		
		(+) AP-1 activity		
		MMP-7 ↑		
		(+) tumor cell invasion		
oral cancer	ApoA-IV	ApoA-IV ↓: (+) tumor progression	screening and diagnostic biomarker	Chang et al. (2019b)
nasopharyngeal carcinoma	ApoA1 ApoE	neutrophil to ApoA1 ratio ↑	neutrophil to ApoA1 ratio	Xue et al. (2020a); Li et al. (2021c)
		OS and RRFS ↓	prognostic biomarker	
		ApoE ↑	for 5-year OS	
		(+) tumor cell invasion	ApoE	
		(+) tumor cell proliferation	diagnostic biomarker	
		(+) tumor cell migration		
larynx squamous cell carcinoma	ApoM ApoL1	ApoM ↑	ApoL1: diagnostic biomarker	Xue et al., 2020b; Zhong et al. (2020)
		(-) tumor cell proliferation		
		(-) tumor cell migration		
		ApoL1 ↑		
		(+) tumor progression		
Esophageal basaloid squamous cell carcinoma	ApoA1	ApoA1 ↓: OS and RFS ↓	independent predictor for OS and RFS	Feng et al. (2021)
Malignant biliary tumor	ApoB	ApoB ↑: OS ↓	prognostic biomarker	Sun et al. (2020)
endometrial carcinoma	ApoE		APOE E2 allele: (+) concurrent occurrence of	Ivanova et al. (2015)
			endometrial hyperplasia and	
			endometrial carcinoma	
ovarian cancer	ApoE	ApoE ↓	biomarker for prediction of chemoresistance	Lai et al. (2018); Zhang et al. (2019c)
		(+) FAK/ERK signal		
		(+) ECM accumulation		
		(+) tumor cell invasion		
		(+) chemoresistance		
acute myeloid leukemia	ApoC2	ApoC2 ↑	ApoC2/CD36: therapeutic target	Zhang et al. (2020)
		(+) ApoC2/CD36/ERK signal		
		(+) leukemia growth		
		OS ↓		
multiple myeloma	ApoA1	ApoA1 ↑: OS ↑	independent prognostic indicator	Liang et al. (2019)

(+) indicates promote (-) indicates inhibit; ↑ indicates increase; ↓ indicates decrease; Apo, Apolipoprotein; ECM, extracellular matrix; ERK, extracellular signal-regulated kinase; c-Jun, AP-1, Transcription Factor Subunit encoded by proto-oncogene JUN; JNK, c-Jun N-terminal kinase; AP-1, Activator protein one; MMP, matrix metalloproteinase; FAK, focal adhesive kinase; OS, overall survival; RRFS, Regional recurrence-free survival; RFS, Recurrence-free survival.

## Application of APOs in cancer treatment

### Targeted therapy

ApoA1 and ApoL1 are tumor suppressors in CRC and thereby scientists are trying to activate the expression of these proteins to inhibit tumor cells. It has been shown that ginsenoside Rp1 (G-Rp1), a type of ginsenoside derived from ginsenoside Rk1, can trigger the over-expression of ApoA1 in CRC cell lines, which promotes apoptosis and prevents proliferation of the tumor cells (Kim et al., 2014). Vitexin can bind to the DNA-binding domain of Heat shock transcription factor-1(HSF-1), inactivating the latter. This fosters the activation of c-Jun N-terminal kinase (JNK) signaling and upregulation of ApoL1, which contributes to autophagic cell death (Bhardwaj et al., 2017). In many solid tumors, liver-X nuclear receptor (LXR) agonism induces the expression of ApoE which then binds to LRP8 on the surface of myeloid-derived suppressor cells (MDSCs), a type of immunosuppressive innate cells. This represses the survival of MDSC and thereby boosts T cell activation and anti-tumor effects (Tavazoie et al., 2018). In contrast, in AML, ApoE mediates immunosuppression to promote cancer development. Therefore, breaking the interaction of ApoE with its downstream effectors may help to enhance immunity against AML. It is found that ApoE can bind to LILRB4, an immune inhibitory receptor, which activates the downstream signaling leading to T cell suppression. Furthermore, h128-3, a specific humanized antibody targeting LILRB4, significantly reverses T cell suppression and AML cell infiltration, and promotes antibody-dependent. Cellular phagocytosis and cellular cytotoxicity (Gui et al., 2019).

Several studies also indicate that microRNA (miRNAs) modulate APOs expression for cancer treatment. MiR-4510 is down-regulated in gastrointestinal stromal tumor (GIST) tissues while its over-expression can suppress the transcription and expression of ApoC2, inhibiting tumor proliferation (Chen et al., 2020b). MicroRNA-30c analogs decrease ApoB expression in hepatoma cells, possibly preventing HCC tumorigenesis as ApoB is a risk factor associated with poorer survival in HCC patients (Yadav et al., 2022).

### APO mimetic peptide in cancer treatment

APO mimetic peptides are designed to mimic structural and functional features of APOs, which show the therapeutic potential for cancer. APO mimetic peptides have been explored to find out new methods for cancer therapy. It is reported that ApoA1 mimetic peptides can reduce viability and inhibit the invasion of ovarian cancer cells. It can also inhibit AKT signaling pathways and increase the sensitivity of ovarian cancer cells to cisplatin, which shows anti-tumor and chemo-sensitive effects (Marinho et al., 2018). ApoA1 mimetic peptide D-4F can inhibit the proliferation and viability of ovarian cancer cells in mice by increasing the expression of the antioxidant enzyme MnSOD. The anti-tumor effect of D-4F is mediated by reducing the cellular oxidative stress and improving the expression and activity of MnSOD (Ganapathy et al., 2012). Another ApoA1 mimetic peptide L-5F can inhibit VEGF- and basic fibroblast growth factor (bFGF)-induced viability, proliferation, invasion, angiogenesis and migration of ovarian cancer cells in a dose-dependent manner. Furthermore, L-5F can also suppress VEGF- and bFGF-induced activation of VEGFR2 and FGFR1 and downstream signaling pathways including AKT and ERK1/2. Injection of L-5F in mice results in a reduced size and quantity of vessels in tumors and decreased level of VEGF in tumor and circulation, indicating its role in inhibiting tumor angiogenesis (Gao et al., 2011).

ApoA1 mimetic peptide L-4F can suppress the progression of pancreatic cancer by repressing inflammation. Specifically, L-4F can inhibit the infiltration of pro-inflammatory cells, especially M2 macrophages, to diminish the secretion of pro-inflammatory cytokines. L-4F can also inhibit the STAT3 and MAPK signaling pathways to prevent the differentiation of M2 macrophage, which further regulates the tumor microenvironment and exerts an anti-inflammatory effect (Peng et al., 2017). In addition, it is found that L-4F can suppress the role of granulocytic myeloid-derived suppressor cells (PMN-MDSCs), increase the production of IFN-γ and TNF-β, and promote the infiltration of CD4^+^ T and CD8^+^ T cell in spleen and pancreatic tumor tissue (Peng et al., 2020). These studies reveal the ability of L-4F to enhance the immune system to kill tumor cells.

In addition to ovarian and pancreatic cancer, ApoA1 can also be a therapeutic strategy for other cancers. It is indicated that ApoA1 mimetic peptide Tg6F can decrease the level of oxidized phospholipid and 25-hydroxycholesterol (25-OHC), which further activates the Notch signaling pathway and reduces the expression of osteopontin (Spp1). This results in the upregulation of patrolling monocytes and downregulation of myeloid-derived suppressor cells (MDSCs) in the lung, inhibiting the tumor formation and progression (Chattopadhyay et al., 2018). Cedó et al. utilized mammary tumor virus-polyoma middle T-antigen transgenic (PyMT) mice to investigate the effect of D-4F treatment to the inherited breast cancer (Cedó et al., 2016). The results show that D-4F can lower the level of plasma oxidized low-density lipoprotein (oxLDL) and inhibit oxLDL-mediated proliferation of MCF-7 cells in mice (Cedó et al., 2016).

### APOs nanoparticles in cancer treatment

In recent years, nanoparticles (NPs) have been considered a hot research area prospectively applied in cancer theranostics, merging the delivery of agents with both anticancer therapeutic effects and diagnostic function (Yetisgin et al., 2020). Due to their unique properties in the nanoscale, their advantages in specifically targeted therapeutic effect on cancer cells have attracted great importance to scientists (Li et al., 2017; Yetisgin et al., 2020). To realize this targeted delivery process, specific ligands bind to their receptors only expressed or over-expressed in cancer cells, boosting the whole anticancer outcome and reducing the side effects caused by the high dose of drugs. Based on the physiological function of APOs and their roles in cancer, NPs modified by the specific type of APOs have been designed to bind to corresponding receptors over-expressed in the specific type of cancer and deliver different anticancer agents.

In the process of constructing efficient nanocarriers to deliver other therapeutic agents, ApoA-I of HDL-based NPs have been applied as a targeted ligand binding to the scavenger receptor B type I (SR-B1) up-regulated in many types of cancer cells to generate endocytosis (Delk et al., 2021). Jiang et al. reported a type of peptide-targeted NPs based on reconstituted high-density lipoprotein (rHDL) through modifying ApoA-I on the surface of hydrophilic shell interacting with SR-B1 overexpressed in triple-negative breast cancer (TNBC) cells. In this nanoplatform, the combination of therapeutic agents in the hydrophobic core was efficiently delivered to cancer cells, including GANT61, an inhibitor of the sonic hedgehog (SHH) signaling pathway, and paclitaxel, a conventional chemotherapeutic drug to inhibit the proliferation of cancer cells. In a series of *in vitro* and *in vivo* experiments, this nanoplatform display satisfactory tumor-specific distribution and therapeutic outcome in which the growth of the primary tumor is significantly inhibited, and the number of metastatic nodules in lungs is drastically reduced (Jiang et al., 2021b). Furthermore, this type of nanoplatform reduces multidrug resistance (MDR) by promoting the uptake of small-molecule chemotherapeutic drugs and inducing fewer adverse effects (An et al., 2021). Gong et al. also developed a drug delivery nanocarrier based on the interaction of ApoA-I mimetic peptide on the synthetic high-density lipoprotein and SR-B1 to boost the uptake of docetaxel by breast cancer cells (Gong et al., 2019). In addition, this delivery strategy is applied in a similar nanoplatform by using ApoA-I to deliver soluble intracellular-acting therapeutic proteins cytochrome C (cytC) conjugated on the surface of NPs into lung cancer cells, inducing targeted apoptosis and tumor growth retardation (Kim et al., 2012). Apart from traditional ApoA-I used in the delivery nanoplatform, mono-cholesterol glutarate (MCG) modified ApoA-I is introduced to a novel stabilized d-rHDL to enhance the capacity of the blood-brain barrier (BBB) penetration and glioma targeting by suppressing the drug leakage (Li et al., 2020b).

LDL-based NPs have become promising drug carriers against cancers by using ApoB as the essential component of LDL interacting with LDLR highly expressed in cancer cells to trigger the uptake of LDL through LDLR-mediated endocytosis (Goldstein et al., 1985; Knott et al., 1985). After transporting LDL into the acidic endosomal–lysosomal system for degradation, the therapeutic agents are intracellularly released to play their roles in full-functioning anticancer effect (Goldstein et al., 1985; Knott et al., 1985; Pires et al., 2012; Huang et al., 2016). Additionally, reconstituted ApoB lipoparticles perform efficiently target delivery with the property of pH-sensitive release and increased cycle time (Yang et al., 2021). Li et al. designed a nanostructured lipid carrier (NLC)-based nanoplatform loaded with doxorubicin prodrug, which displays higher tumor cell uptake and inhibition *in vitro* experiments, and satisfactory anti-orthotopic breast cancer activity and lower systemic side effects *in vivo* studies (Li et al., 2019).

ApoE has been regarded as another vital targeting ligand to specifically transport therapeutic agents into cancer cells, mainly through binding to LDLRs over-expressed on glioma cells and hepatocellular carcinoma (HCC) (Kang et al., 2016b; Oller-Salvia et al., 2016). Furthermore, ascribed to the physiological function of ApoE to cross biological barriers such as BBB, it has been widely used in nanoplatforms against brain-related cancers (Kreuter et al., 2002; Re et al., 2011). In the targeted delivery of chemotherapeutic agents, the lipid-binding domain of ApoE can be connected to paclitaxel decorated with amyloid β-protein (Aβ)-CN peptide to form protein corona exposing the receptor-binding domain outside for targeted delivery (Zhang et al., 2021). In addition to chemotherapy, the brain-targeted delivery of small molecule inhibitors has been performed in a similar strategy, including Bcl-2/Bcl-xl and Mcl-1 inhibitors to activate mitochondrial-mediated cell apoptosis and RAS inhibitor to block oncogenic signaling pathways (Qin et al., 2019; He et al., 2022b). After delivering the brain-targeted nanoplatform modified by ApoE peptide and pH-sensitive dextran, the targeted release of dual inhibitors containing Bcl-2 family (Bcl-2 and Bcl-xl) inhibitor ABT-263 and Mcl-1-specific inhibitor A-1210477 (A12) exhibits the capacity of efficient BBB penetration and good biocompatibility for synergistic anti-GBM effect (He et al., 2022b). Similarly, rigosertib (RGS), a RAS effector protein inhibitor crossing BBB *via* transcytosis, is released in the reduced intracellular environment mediated by ApoE-derived peptide-targeted chimeric polymersomes (ApoE-CP), which inhibits cell proliferation through G2/M cell cycle arrest and apoptosis of GBM cells (Qin et al., 2019). Gene therapy has been improved by ApoE-mediated brain-targeted delivery of oligonucleotide miRNA inhibitors (OMIs) to inactivate dysregulated miRNAs (Grafals-Ruiz et al., 2020). As an emerging anticancer therapy, noninvasive photothermal therapy (PTT) offers higher therapeutic efficiency through brain-targeting ApoE peptide-based NPs for the delivery of a novel photosensitizer with the property of aggregation-induced-emission under the excitation of near-infrared IIb (Wang et al., 2022). ApoE-mediated systemic nanoplatform also elevates the BBB permeability and targetability of immunotherapy to deliver a mediator of natural killer cells and cytotoxic T lymphocytes called granzyme B (GrB). It facilitates to attack mitochondria and release cytochrome C, killing glioma cells. In this process, specific tumor antigens are released to induce immunogenic cell death (ICD), which further promotes immunotherapeutic outcomes with the assistance of immunoadjuvants NPs consisting of ApoE and CpG oligonucleotide (CpG) to achieve ApoE peptide-mediated glioma codelivery of GrB and CpG for potent systemic immunotherapy (Wei et al., 2022).

In the application of APOs in NPs based-anticancer therapy, APOs primarily function as a targeted ligand to facilitate the tumor-specific delivery of various therapeutic agents associated with chemotherapy, targeted inhibitors, gene therapy, PTT, and immunotherapy. Based on the biological characteristics of different types of cancer, corresponding APO is conjugated in related NPs to cross specific barriers and target cancer cells over-expressed specific receptors. These delivery tactics also provide new ideas for transporting other novel therapeutic agents and the combination of multiple therapies for synergistic effects in the future.

## Conclusion

**FIGURE 3:**
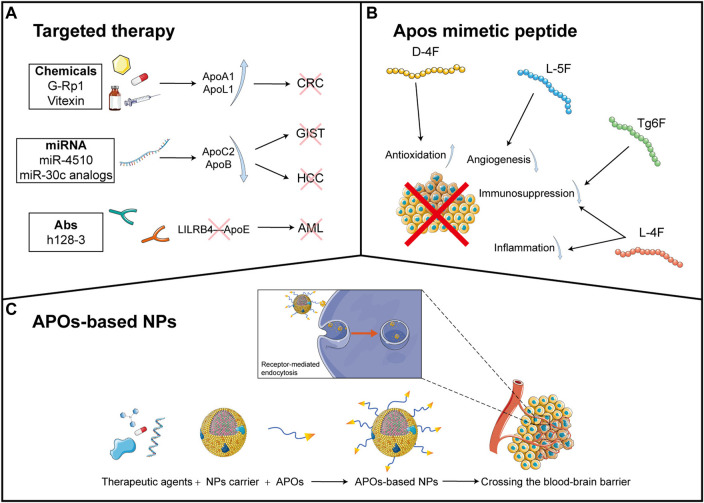
APO-related anti-cancer therapies **(A)**: The current anti-cancer therapeutic agents targeting APOs are divided into three types: chemicals, miRNA and antibodies. Chemicals such as G-RP1 and vitexin induce the upregulation of ApoA1 and ApoL1, respectively, which inhibit CRC proliferation. miRNA agents include miR-4510 and miR-30c analogs, which downregulate the expression of ApoC1 and ApoB respectively. These eventually hamper the development of GIST and HCC. Antibodies such as h128-3 can bind LILRB4 and thereby disrupt the interaction of LILRB4 and ApoE, preventing the progression of AML (G-Rp1: Ginsenoside Rp1; CRC: colorectal cancer; miRNA: microRNA; GIST: gastrointestinal stromal tumor; HCC: hepatocellular carcinoma; AML: acute myeloid leukemia.) **(B)**: Apolipoprotein mimetic peptides show anti-tumor effects. D-4F induces antioxidation while L-5F induces angiogenesis. Both L-4F and Tg6F promote immunosuppression, and L-4F also facilitates inflammation. All of these events trigger tumor-killing effects **(C)**: The general components of APOs-based NPs include therapeutic agents in the core of NPs, NPs carrier, and APOs conjugated on the surface of NPs. The therapeutic agents include chemotherapeutic drug (the capsule), small molecular inhibitor (the irregular polygon), oligonucleotide miRNA inhibitor (the single strand nucleotide chain), and photosensitizer (the chemical structural formula). Due to the ability of APOs to bind to receptors of some biological barrier and cells, the NPs can cross the blood-brain barrier (BBB) and trigger the receptor-mediated endocytosis to facilitate the targeted delivery of therapeutic agents into cancer cells, achieving enhanced anticancer outcome (NP: Nanoparticles). This figure is partially made using Servier Medical Art (smart.servier.com).

This review summarized the current findings of APOs in cancers, including the potential mechanisms, their roles in predicting cancer diagnosis and progression, and their genetic polymorphisms associated with cancer susceptibility in certain populations. APOs exert anti- or pro-tumor effects depending on the type of cancer and different APO members act distinctively *via* regulating classical signaling pathways in cancers and cell components in tumor microenvironment. Additionally, we also concluded the potential therapeutic strategies based on APOs, which includes APO targeted therapy, APOs mimetic peptides and APOs-based nanoparticles (Figure 3). Thus, modulation of APOs may shed light on novel anti-cancer therapies. We hope that our review may provide new insight into the involvement of APOs in cancers and their applications in anti-cancer therapies.
